# mRNA Expression of *VEGF* and Its Receptors in Fallopian Tubes of Women with Ectopic Pregnancies 

**DOI:** 10.22074/ijfs.2015.4209

**Published:** 2015-04-21

**Authors:** Nafise Zarezade, Samane Saboori Darabi, Fariba Ramezanali, Elham Amirchaghmaghi, Gholamreza Khalili, Ashraf Moini, Reza Aflatoonian

**Affiliations:** 1 Department of Endocrinology and Female Infertility at Reproductive Biomedicine Research Center, Royan Institute for Reproductive Biomedicine, ACECR, Tehran, Iran; 2 Department of Biochemistry, Payame Noor University, Tehran, Iran; 3 Department of Epidemiology and Reproductive Health at Reproductive Epidemiology Research Center, Royan Institute for Reproductive Biomedicine, ACECR, Tehran, Iran; 4 Department of Gynecology and Obstetrics, Arash Women's Hospital, Tehran University of Medical Sciences, Tehran, Iran; 5 Vali-e-Asr Reproductive Health Research Center, Tehran University of Medical Sciences, Tehran, Iran

**Keywords:** Ectopic Pregnancy, Fallopian Tube, Vascular Endothelial Growth Factor, VEGF Receptor, Gene Expression

## Abstract

**Background:**

Establishment of viable pregnancy requires embryo implantation and placentation. Ectopic pregnancy (EP) is a pregnancy complication which occurs when an embryo implants outside of the uterine cavity, most often in a fallopian tube. On the other hand, an important aspect of successful implantation is angiogenesis. Vascular endothelial growth factor (VEGF) is a potent angiogenic factor responsible for vascular development that acts through its receptors, VEGF receptor 1 (VEGFR1) and VEGFR2. This study aims to investigate mRNA expression of *VEGF* and its receptors in fallopian tubes of women who have EP compared with fallopian tubes of pseudo-pregnant women. We hypothesize that expression of *VEGF* and its receptors in human fallopian tubes may change during EP.

**Materials and Methods:**

This was a case-control study. The case group consisted of women who underwent salpingectomy because of EP. The control group consisted of women with normal fallopian tubes that underwent hysterectomy. Prior to tubal sampling, each control subject received an injection of human chorionic gonadotropin (hCG) to produce a state of pseudo-pregnancy. Fallopian tubes from both groups were procured. We investigated *VEGF, VEGFR1* and *VEGFR2* mRNA expressions in different sections of these tubes (infundibulum, ampulla and isthmus) by reverse transcription polymerase chain reaction (RT-PCR) and quantitative PCR (Q-PCR).

**Results:**

RT-PCR showed expressions of these genes in all sections of the fallopian tubes in both groups. Q-PCR analysis revealed that expressions of *VEGF, VEGFR1* and *VEGFR2* were lower in all sections of the fallopian tubes from the case group compared to the controls. Only *VEGFR2* had higher expression in the ampulla of the case group.

**Conclusion:**

Decreased expressions of *VEGF, VEGFR1* and *VEGFR2* in the EP group may have a role in the pathogenesis of embryo implantation in fallopian tubes.

## Introduction

An ectopic pregnancy ( EP ) is a pregnancy complication which occurs when an embryo implants anywhere other than the intrauterine cavity. EP is a major cause of maternal morbidity and mortality in the first trimester of pregnancy (1,3) and its incidence has increased in the last two decades (4). Several risk factors for EP have been identified including pelvic inflammatory disease ( PID ) following sexually transmitted diseases ( STDs ), damage and infection of the fallopian tubes, endometriosis, history of tubal surgery and previous EP. More than 95% of EPs occur in the fallopian tubes ( tubal pregnancy ) (5). The majority are located in the ampulla ( 80% ), followed by the isthmus and infundibulum, respectively (6). 

Establishment of a viable pregnancy requires implantation and placentation, both important and critical processes (7). An important aspect of successful implantation is the organization of angiogenesis which is mediated by a number of growth factors (8). Vascular endothelial growth factor ( VEGF ) is a potent angiogenic factor produced by different tissues of the female reproductive tract including the endometrium, ovaries (9,10), trophoblast and corpus luteum (10,11). VEGF is a major modulator of vascular growth, remodeling and permeability in the endometrium, decidua, and trophoblast. It is responsible for vascular development in the embryo (11,15). In addition, VEGF stimulates endothelial cell proliferation, promotes cell migration and inhibits apoptosis. In mammals there are five isoforms of the VEGF family: VEGFA, B, C, D and placental growth factor ( PLGF ) (16). These isoforms are produced as a result of alternative splicing from VEGF mRNA (17). VEGFs act on endothelial cells through their receptors. These receptors include three receptor protein-tyrosine kinases ( VEGFR1, VEGFR2 and VEGFR3 ) and two non-protein kinase receptors ( neuropilin-1 and -2 ) (18). Different cytokines, growth factors and gonadotropins modulate VEGF expression. Factors such as fibroblast growth factor-4 ( FGF-4 ) (19), platelet-derived growth factor ( PDGF ) (20), tumor necrosis factor-α ( TNF-α ) (21), transforming growth factor-β ( TGF-β ) (22), keratinocyte growth factor ( KGF ) (23), insulin-like growth factor-I ( IGF-I ) (24), interleukin-1β ( IL-1β ) (25) and IL-6 (26) result in up-regulation of VEGF expression. In addition, hypoglycemia and hypoxia are two important inducers of VEGF expression (27). 

Fallopian tubes play an essential role in successful human reproduction. They provide an appropriate environment for pre-implantation development of the embryo and its transportation to the uterus (28). Previously, Lam et al. (29,30) have investigated the expression of *VEGF* and its receptors ( *VEGFR1* and *VEGFR2* ) in normal human fallopian tubes and reported expressions of *VEGF, VEGFR1* and *VEGFR2* in various regions of the fallopian tubes of fertile women. 

On the other hand, *VEGF* plays important roles in embryo implantation. Several investigators (13,15) have reported that *VEGF* expression in the endometrium and corpus luteum may be regulated by ovarian steroids and possibly beta human chorionic gonadotropin ( β-hCG ). Evans et al. (31) report that *VEGF* levels increase during the ﬁrst trimester of a normal pregnancy and show a positive correlation with gestational age as well as β-hCG, estrogen ( E2 ), and progesterone levels. Although some studies focused on roles of *VEGF* and its receptors in important gynecological diseases and pregnancy complications including endometriosis (15,32,33), recurrent abortion (34,36) and EP (37), more details need to be investigated. The current research aims to study mRNA expressions of *VEGF* and its receptors in fallopian tubes with EP compared with fallopian tubes of pseudo-pregnant women as the control group. 

## Materials and Methods

### Tissue collection for genomic studies

All procedures were approved by the Ethics Committee of Royan Institute. Written informed consent was obtained prior to sample collections. All specimens were collected at the Arash Womenʼs Hospital, Tehran, Iran. 

### Case group

In this case-control study, ten fallopian tubes were obtained from women who underwent salpingectomy because of EP. 

### Control group

Due to inaccessibility to normal fallopian tubes of pregnant women as the control group, we decided to investigate the fallopian tubes obtained from women who underwent hysterectomy because of benign gynecological conditions that did not affect the tubes. All women in the control group were fertile and had regular menstrual cycle with no evidence of any pathological tubal disorders. To induce hormonal conditions similar to a normal pregnancy, each woman from the control group received intramuscular injections of 5000 IU per injection of hCG. For this purpose, hCG was administered every 3 days beginning in the midluteal phase before total abdominal hysterectomy, for a 12-day period. This treatment creates a state of pseudo-pregnancy which is harmless and has been previously used by other research studies (38,39). 

### Sampling and processing

In order to ensure the integrity of tubal morphology and function in the case group, we excised at least 1 cm away from the implantation site of the embryo in the fallopian tube to avoid collection of any embryonic or trophoblastic tissues. 

After obtaining required samples from both case and control groups we cut each fallopian tube into three regions infundibulum, ampulla and isthmus. Then, each of these regions was divided into small ( 1×1 cm ) pieces and immediately placed in RNAlater solution ( Ambion, Huntington, UK ). In the next step, samples immediately immersed in liquid nitrogen and stored at -80˚C until processing. 

### Total RNA extraction, cDNA synthesis and reverse transcription polymerase chain reaction ( RT-PCR )

Samples were removed from RNAlater and TRI reagent ( Sigma, Pool, UK ) was used for total RNA isolation according to the manufacturer's instructions as used in our previous study (40). RNA concentrations were quantified by spectrophotometric analysis. 

First-strand cDNA synthesis was performed using oligodT primers and the Superscript II Reverse Transcriptase System ( Fermentas, Sanktleon-rot, Germany ). Briefly, reverse transcription was performed according to the recommended method ( incubation for 60 minutes at 42˚C and termination of the reaction by heating at 70˚C for 5 minutes ). cDNA was amplified by RT-PCR using the prepared cDNA, forward and reverse primers of human *VEGF, VEGFR1, VEGFR2* and *β-actin* ( Metabion, Martinsried, Germany ), and Platinum Blue PCR Super Mix ( Invitrogen, Paisley, UK ). Primer sequences used in this study are shown in table 1. *β-actin* was used as the housekeeping gene. 

Cycling conditions were continued for 40 cycles as follows: 5 minutes at 95˚C for initial denaturation, followed by 40 cycles of 45 seconds at 95˚C, 45 seconds at 60˚C and 45 seconds at 72˚C. Reactions were also amplified in the absence of reverse transcriptase as negative RT controls. Human placenta samples were used as the positive control (41) while non template water was used as the negative control. PCR products were subjected to electrophoresis on 1.7% agarose gel ( Sigma, UK ) that contained ethidium bromide and visualized under ultraviolet ( UV ) light to capture image. 

### Quantitative PCR (Q-PCR)

Q-PCR was used to quantify whether there was any difference in *VEGF* and *VEGFRs* mRNA expression levels in fallopian tubes of the case and control groups. In this procedure we used the prepared cDNA, Power SYBR Green Master Mix ( Applied Biosystems, UK ) and primers of human *VEGF, VEGFR1, VEGFR2* and *β-actin* (Table 1). All experiments included negative controls with no cDNA. Each reaction of the PCR plate contained 10 µl SYBR green, 6 µl molecular grade water, 1 µl of each forward and reverse primers and 2 µl cDNA. The amplification was performed under the following conditions: 10 minutes at 95˚C, 50 cycles at 95˚C for 15 seconds and 60˚C for 60 seconds. Q-PCR was performed under standard conditions and all experiments were run in triplicate. Relative *VEGF* and *VEGFRs* expression quantities were compared between case and control groups. Q-PCR data were analyzed using the comparative cycle threshold ( CT ) method (42). The difference in CT ( ∆CT ) was determined as the difference between the number of cycles required for ampliﬁcation of the test gene and the housekeeping gene, human *β-actin*. Then ∆∆CT was calculated by ﬁnding the difference between case and control groups. 

Differences in normalized expression values between groups were analyzed using the analysis of variance ( ANOVA ) statistical test. The results were presented as mean±standard error of mean ( SEM ). The level of statistical significance was set at p<0.05. 

## Results

### Reverse transcription polymerase chain reaction (RT-PCR)

This study enrolled 20 women, 10 for the control and 10 for the case groups. The mean age of the control group was 47.5±5.36 years which signiﬁcantly differed from the case group ( 36±5.69 years ). The age range was 42-56 years old for the control group and 26-40 years for the case group. 

**Fig.1:**
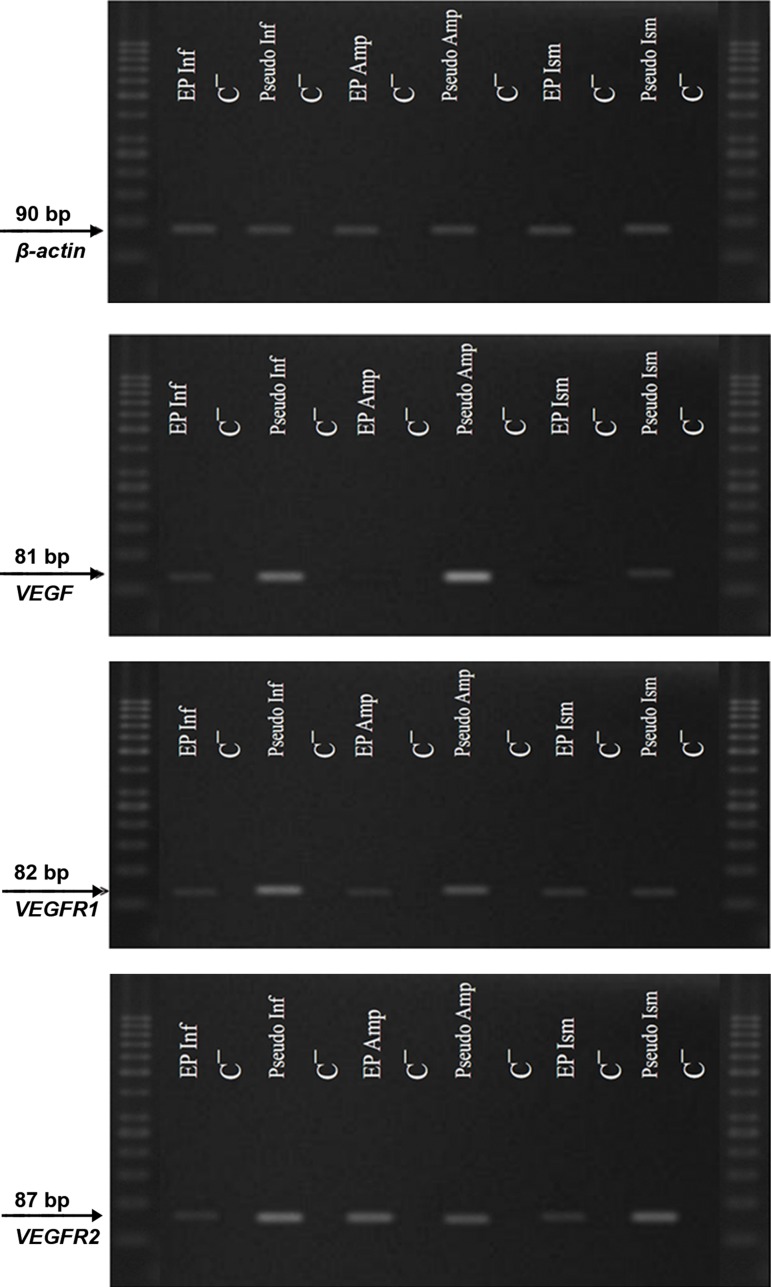
Expressions of vascular endothelial growth factor (VEGF) and its receptors, VEGF receptor 1 (VEGFR1) and receptor 2 (VEGFR2) mRNA in the infundibulum (Inf), ampulla (Amp) and Isthmus (Ism) of fallopian tubes. These genes were expressed in all parts of the fallopian tubes in both case and control groups. There was no amplified product in negative control (C¯) samples.

RT-PCR showed that *VEGF* and *VEGFRs* mRNA were expressed in all regions of the fallopian tubes ( infundibulum, ampulla and isthmus ) in both case and control groups (Fig .1). All ampliﬁed products were at the predicted size of their respective genes. There was no ampliﬁed product in negative control samples which was indicative of the absence of genomic DNA contamination. 

### Quantitative PCR (Q-PCR)

**Fig.2:**
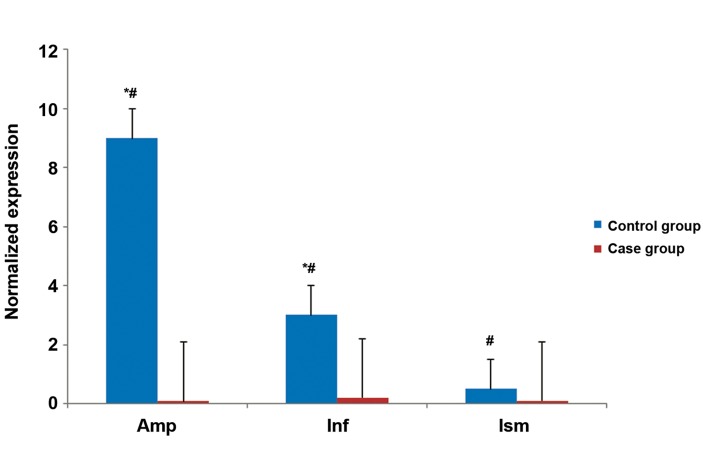
Quantitative PCR (Q-PCR) results of vascular endothelial growth factor (VEGF) mRNA expression. *; Significantly different expression between the two groups, #; Significantly different expression between different parts of the control group, Amp; Ampulla, Inf; Infundibulum and Ism; Isthmus. The level of statistical significance was set as at p<0.05.

**Fig.3:**
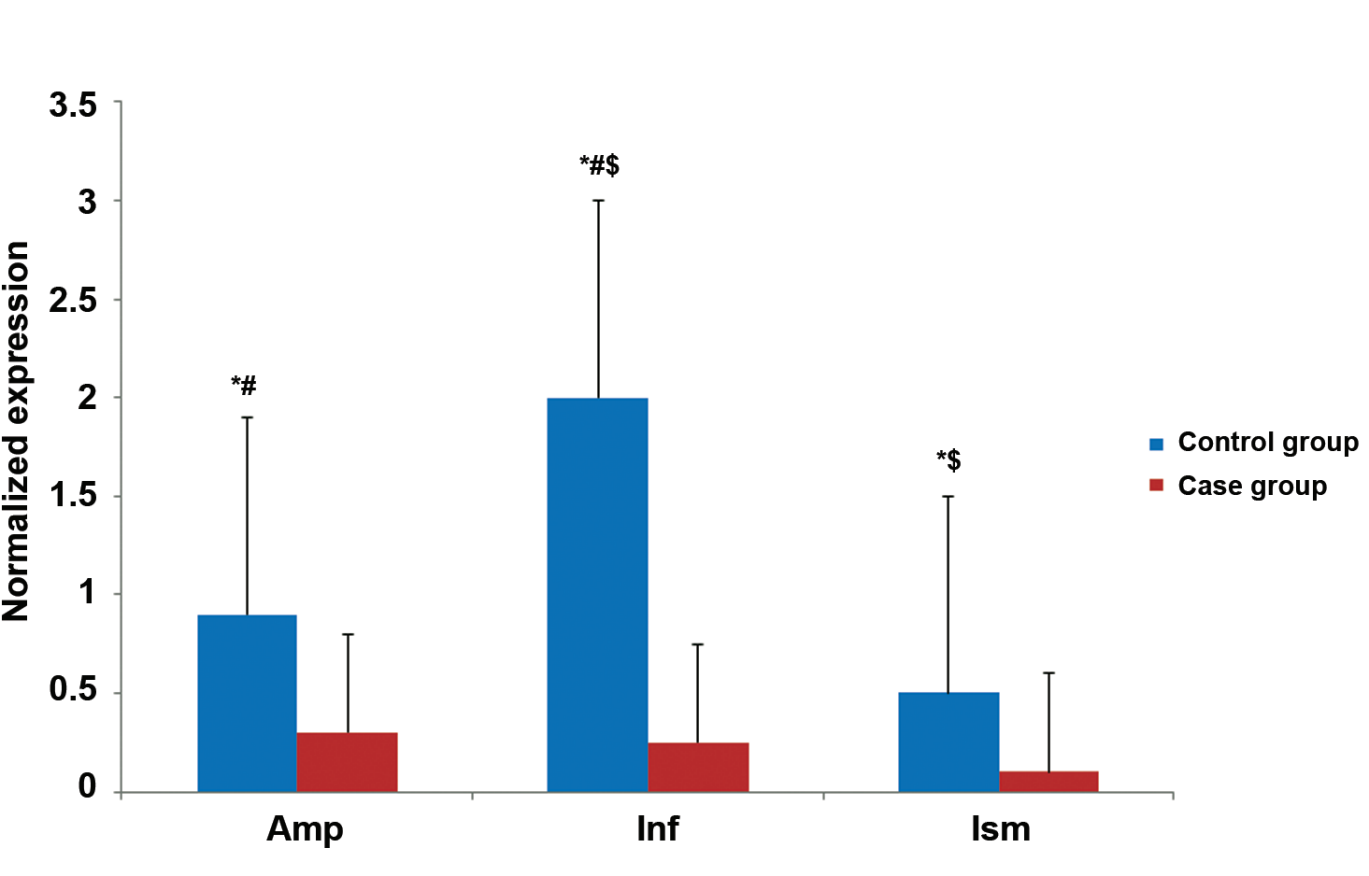
Quantitative PCR (Q-PCR) results of vascular endothelial growth factor receptor 1 (VEGFR1) mRNA expression. *; Significantly different expression between the two groups, #; Significantly different expression between the ampulla and infundibulum of the control group, $; Significant different expression between the isthmus and infundibulum of the control group, Amp; Ampulla, Inf; Infundibulum and Ism; Isthmus. The level of statistical significance was set as at p<0.05.

**Fig.4:**
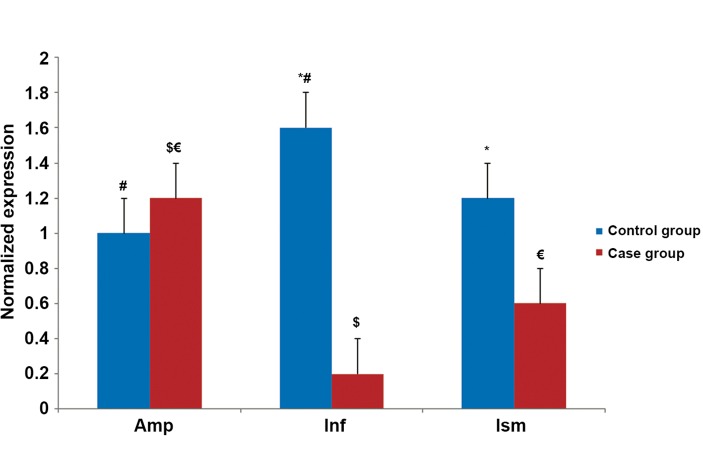
Quantitative PCR (Q-PCR) results of vascular endothelial growth factor receptor 2 (VEGFR2) mRNA expression. *; Significantly different expression between two groups, #; Significantly different expression between the ampulla and infundibulum of the control group, $; Significantly different expression between the ampulla and infundibulum of the case group, €; Significantly different expression between the ampulla and isthmus of the case group, Amp; Ampulla, Inf; Infundibulum and Ism; Isthmus. The level of statistical significance was set as at p<0.05.

The quantitative expression profiles of *VEGF, VEGFR1* and *VEGFR2* in all regions of the fallopian tubes in both groups are shown in figures 2-4. The results revealed that expressions of all studied genes differed between the two groups and were significantly higher in the pseudo-pregnant women (control group) compared to the EP women (case group). As an exception, we observed higher *VEGFR2* gene expression in the ampulla of the case group.

The expression of *VEGF* was the highest in the ampulla of the control group and infundibulum of the case group compared to the other areas of the same tube (Fig.2). In contrast to *VEGF, VEGFR1* and *VEGR2* expressions were highest in the ampulla of the case group and infundibulum of the control group (Figs.3, 4).

**Table 1:** The sequences of primers used to amplify genes of interest


Gene	Forward primer (5΄-3΄)	Reverse primer (5΄-3΄)	Annealing temperature (˚C)	Product size (bp)	GenBank accession no.

**VEGF**	TGCAGATTATGCGGATCAAACC	TGCATTCACATTTGTTGTGCTGTAG	60	81	AB021221
**VEGFR1**	CAGGCCCAGTTTCTGCCATT	TTCCAGCTCAGCGTGGTCGTA	60	82	AF063657
**VEGFR2**	CCAGCAAAAGCAGGGAGTCTGT	TGTCTGTGTCATCGGAGTGATATCC	60	87	AF063658
**β-actin**	CAAGATCATTGCTCCTCCTG	ATCCACATCTGCTGGAAGG	60	90	NM 001101

VEGF; Vascular endothelial growth factor, VEGFR1; VEGF receptor 1, VEGFR2; VEGF receptor 2 and ß-actin; Beta actin.

## Discussion

Embryo implantation is an essential process that occurs in the early stage of pregnancy and leads to the establishment of a functional placenta and pregnancy. Successful embryo implantation depends on proper interactions between the blastocyst and a receptive endometrium. For development of a receptive endometrium, ovarian hormones that include estrogen and progesterone act on epithelial cells of the endometrium leading to the establishment of an appropriate environment which supports blastocyst development, attachment and subsequent implantation events (43,45). The processes of implantation and trophoblast invasion are associated with growth of blood vessels coincident with decidualization, improvement of vascular membranes, and placenta formation (46). These processes accompanied by the formation of new blood vessels from pre-existing vasculature ( angiogenesis ) (47) and establishment of the embryonic vascular system ( vasculogenesis ) (48). 

One of the key factors in regulation of angiogenesis is VEGF. It has been suggested that VEGF is an essential cytokine for embryo implantation. This cytokine plays a crucial role in maternalfetal interactions as a local mediator which facilitates blastocyst implantation (30). Expressions of VEGF and its receptors are induced by growth factors, cytokines and gonadotropins, and depend on local conditions such as hypoxia (27). 

Although many cellular and molecular events during embryo implantation are unknown, studying of these changes during normal human pregnancy is practically impossible because of ethical limitations. On the other hand, most women with EP undergo salpingectomy as treatment. Thus, EP can be used as an accessible model for human embryo implantation. 

In the present study we investigated mRNA expression levels of *VEGF* and its receptors in fallopian tubes of women with EP ( case group ) compared to a control group ( pseudo-pregnant women with normal fallopian tubes ). Because of ethical limitations, accessibility to fallopian tubes of normal pregnant women is impossible; therefore we have injected hCG for the induction of pseudopregnant conditions in control group women that underwent hysterectomy (5). RT-PCR showed that VEGF and VEGFRs mRNA expressed in all regions of the fallopian tubes of both groups. QPCR conﬁrmed that the relative expression of these genes was signiﬁcantly higher in fallopian tubes of pseudo-pregnant women compared with fallopian tubes of case group, with the exception of VEGFR2 mRNA expression. 

Previously, other studies investigated the presence of *VEGF* and its receptors in normal fallopian tubes. Lam et al. (29,30) conducted immunohistochemical analysis and showed that VEGF, VEGFR1 and VEGFR2 expressed at the protein level in the infundibulum, ampulla and isthmus of fallopian tubes in fertile women throughout the menstrual cycle. Using semi-quantitative RT-PCR, they observed that VEGF, VEGFR1 and 2 mRNA expressions were highest in fallopian tubes in the periovulatory stage. Expressions in the ampullary and infundibular regions were higher than the isthmus. In addition, they reported a signiﬁcant positive correlation between serum follicle stimulating hormone ( FSH ) and luteinizing hormone ( LH ) concentrations and *VEGF* and *VEGFR1* mRNA expressions in normal fallopian tubes. They hypothesized that *VEGF* in human fallopian tubes might play important roles related to early reproductive events, which occur predominantly in the ampulla during the peri-ovulatory phase when serum FSH and LH concentrations were high. 

The ﬁnding of the present study in pseudo-pregnant women was consistent with another study by Lam et al. (29,30) who observed the highest expressions of *VEGF* and *VEGFR1* in the ampullary and infundibulary regions, respectively. 

A study of *VEGF* family gene expression in EP conducted by Lam et al. (37), investigated the implantation site of fallopian tubes with EP compared to other regions of same fallopian tube. In the present study we excluded the implantation site. In their study (37), *VEGF* and *VEGFRs* mRNA expressions increased at the implantation site of the fallopian tube with the EP compared to the rest of the same fallopian tube. The current study differed from the study by Lam et al. in the samples used for comparison. They compared the expression of these genes at the implantation site and other sections of same fallopian tube with EP. The current study collected fallopian tubes from normal women who received hCG to mimic the hormonal status of a normal pregnancy ( pseudo-pregnant state ) as the control group (38,39). 

Lower gene expression of *VEGF* and its receptors in the EP group compared to pseudo-pregnant women from the control group might be due to the effect of hCG on *VEGF* and *VEGFRs* expressions. The result of a study by Lam et al. (29) showed that mRNA expression of *VEGF* in normal fallopian tubes was positively correlated with serum sex hormone concentrations. 

Another potential explanation for the difference in gene expression between the case and control groups in the present study might be the differences in age between the studied women. The mean age of the control group was 47.5 years old, whereas the case group was 36. The increased *VEGF* and *VEGFRs* mRNA expression in the control group might be secondary to changes in sex hormones, cytokines and growth factor expressions in older women. 

Despite limited studies with regards to *VEGF* gene expression, several studies investigated the concentration of *VEGF* in sera of pregnant women. Evans et al. (31) stated that maternal serum *VEGF* concentrations increased during the ﬁrst trimester of pregnancy. In a study by Wheeler et al. (49) maternal serum *VEGF* concentrations remained elevated up to week-20 of pregnancy and was positively correlated with placental volume at mid-pregnancy, as well as to placental and fetal weight at delivery. Daniel et al. (50) reported that serum *VEGF* levels up-regulated in women with EP compared to those who had intrauterine pregnancies, although there was only borderline signiﬁcance between tubal EP and failed intrauterine pregnancy. 

## Conclusion

Our results suggest that expressions of *VEGF* and *VEGFRs* mRNAs are lower in fallopian tubes that contain EP compared with normal fallopian tubes that receive hCG. Further studies are required with larger sample size that include a group of fertile women in the periovulatory phase. *VEGF VEGFRs* Acknowledgements We would like to thank the staff at Royan Institute for technical assistance as well as the staff at Arash Womenʼs Hospital for recruitment of subjects. This study was ﬁnancially supported by Royan Institute. There was no conflict of interest in this project. 
